# Smooth Muscle Cell—Macrophage Interactions Leading to Foam Cell Formation in Atherosclerosis: Location, Location, Location

**DOI:** 10.3389/fphys.2022.921597

**Published:** 2022-06-20

**Authors:** Pinhao Xiang, Valentin Blanchard, Gordon A. Francis

**Affiliations:** Department of Medicine, Centre for Heart Lung Innovation, Providence Research, St. Paul’s Hospital, University of British Columbia, Vancouver, BC, Canada

**Keywords:** atherosclerosis, smooth muscle cells, macrophages, foam cells, human, mouse

## Abstract

Cholesterol-overloaded cells or “foam cells” in the artery wall are the biochemical hallmark of atherosclerosis, and are responsible for much of the growth, inflammation and susceptibility to rupture of atherosclerotic lesions. While it has previously been thought that macrophages are the main contributor to the foam cell population, recent evidence indicates arterial smooth muscle cells (SMCs) are the source of the majority of foam cells in both human and murine atherosclerosis. This review outlines the timeline, site of appearance and proximity of SMCs and macrophages with lipids in human and mouse atherosclerosis, and likely interactions between SMCs and macrophages that promote foam cell formation and removal by both cell types. An understanding of these SMC-macrophage interactions in foam cell formation and regression is expected to provide new therapeutic targets to reduce the burden of atherosclerosis for the prevention of coronary heart disease, stroke and peripheral vascular disease.

## Introduction

Smooth muscle cells (SMCs) and macrophages form the bulk of the cells in all stages of human and mouse atherosclerosis. Studies since the early 1990’s have mostly used mice to study atherogenesis, and have suggested that this process is primarily macrophage-driven, is a consequence of the endothelial damage caused by the severe dyslipidemia required to induce atherosclerosis in these animals, and that SMCs play a lesser role (Libby, 2021). In contrast, the initial stages of human atherogenesis occur on a background of pre-atherosclerotic thickening of the arterial intima believed to be caused by migration and proliferation of medial SMCs early in life in atherosclerosis-prone arteries, prior to lipid deposition (Allahverdian et al., 2018). These SMCs promote the retention of lipids by secreting negatively-charged proteoglycans that bind positively-charged apolipoprotein B (apoB) on atherogenic lipoproteins, before the presence of many monocytes/macrophages in the lesion. The interactions between monocytes/macrophages and SMCs are therefore dependent both on the species being examined and the stage of atherosclerosis. In this review we outline what is known regarding the timing of appearance and proximity of SMCs and macrophages to each other in different stages of human and mouse atherosclerosis, and how this allows interactions between these cell types. Secondly, we review current knowledge about lipid uptake mechanisms and cellular factors secreted by both SMCs and macrophages that may be involved in the formation of foam cells of each cell type, as well as cholesterol removal from these cells. An understanding of these interactions is expected to identify potential new targets to reduce atherosclerosis for the prevention and treatment of ischemic vascular disease.

## Appearance and Role of Smooth Muscle Cells and Macrophages in Early Human and Mouse Atherosclerosis

Mice are the most widely used animal model for the study of atherosclerosis, due to their short developmental and life cycle, ease of genetic modification, low-cost rearing, small size and high reproductive rate. However, mice and humans exhibit significant genetic and physiologic differences influencing major steps in the process of atherogenesis.

Wild-type (WT) mice do not develop spontaneous atherosclerosis owing in part to their low-fat diet, low plasma cholesterol and overall atheroprotective lipoprotein metabolism (Nakashima et al., 1994). In particular, mice do not express cholesteryl ester transfer protein (CETP), a plasma protein shuttling cholesteryl esters from high-density lipoproteins (HDL) to apoB-containing lipoproteins including very-low- (VLDL), intermediate- (IDL) and especially low-density lipoproteins (LDL). Consequently, while in humans up to 2/3 of total cholesterol is transported in atherogenic apoB-containing lipoproteins, mice carry most of their cholesterol in atheroprotective HDL particles (Gordon et al., 2015). The induction of atherosclerosis in mice thus requires a susceptible mouse strain (C57BL/6) plus extreme pro-inflammatory and hyperlipemic conditions characterized by an exaggerated elevation of plasma cholesterol levels 6–10 times the levels seen in WT mice (Reddick et al., 1994). These inflammatory conditions mean that initiation of atherosclerosis is primarily immune cell/macrophage-driven in mice. Another major difference is the absence in mice of pre-atherosclerotic thickening of the intima by SMCs and their secreted proteoglycans (Diffuse Intimal Thickening, DIT), a critical precursor required for future development of atherosclerosis in human arteries.

These major differences between may lead to inaccurate assumptions of what drives early as well as later stages of atherosclerosis in humans when comparing results with mice. Hereafter, we describe the appearance and pathological mechanisms involving SMCs and macrophages and their interactions from the pre-atherosclerotic stage to the advanced plaque in these two species.

## Distribution of SMCs and Macrophages in Human Atherosclerosis

Atherosclerosis is a disease of medium and large size arteries that progresses from the initial deposition and accumulation of lipids derived from circulating apoB-containing lipoproteins in the already formed DIT layer of SMCs and proteoglycans of atherosclerosis-prone arteries. Nakashima and co-workers described elegantly the chronological stages of pre-atherosclerotic DIT, lipid infiltration and retention, macrophage recruitment, and pathological intimal thickening (PIT) as the last stage before progression to an advanced lesion (Nakashima et al., 2007).

### Initial Lipid Deposition in DIT

Certain mammalian species including humans and non-human primates but not mice, rat or rabbits, exhibit DIT in all atherosclerosis-prone arteries of their vascular system (Stout et al., 1983). The critical role of DIT in atherosclerosis is indicated by the observation that atherosclerotic lesions appear only in arteries exhibiting DIT such as the coronary and carotid arteries and aorta (Nakashima et al., 2002), whereas atherosclerosis-resistant vessels such as the internal thoracic artery lack DIT. DIT devoid of lipid deposition begins in utero, is present in all humans by the age of 2 years (Ikari et al., 1999), and is regarded as a physiological adaptation to changes in artery flow and wall tension (Stary et al., 1994). At this pre-atherosclerotic stage, rare resident patrolling macrophages may be observed near the endothelium but not in deeper layers of the intima (Stary et al., 1992).

SMCs particularly in the deeper intima secrete high levels of the negatively charged proteoglycans biglycan and decorin (Nakashima et al., 2007). According to the response-to- retention hypothesis of atherosclerosis proposed by Williams and Tabas, diffusion of circulating positively-charged apoB-containing lipoproteins into the intima, which is based on non LDL-receptor (LDLR)-dependent mechanisms (Jang et al., 2020), and occurs particularly in the presence of elevated circulating LDL cholesterol (LDL-C) such as in familial hypercholesterolemia, over a lifetime results in their infiltration into and retention in the subendothelial space (Williams and Tabas, 1995). This lipid retention occurs primarily in the deep intima based on the charge-charge interaction between apoB and biglycan and decorin secreted in high amounts by SMCs in this region, and prior to any significant monocyte infiltration in human atherosclerosis (Napoli et al., 1997) (Nakashima et al., 2007). These observations suggest human atherosclerosis occurs primarily as a response to retention of lipids, rather than as a response to injury as in mice.

### Fatty Streak to Pathological Intimal Thickening

Fatty streaks are visually recognizable by the deformation and thickening of the intima towards the arterial lumen, though, in this early stage of atherosclerosis, most of this lipid is deposited in the deep intima surrounding SMCs, as well as being internalized in SMC foam cells (Katsuda et al., 1992; Nakashima et al., 2007). Whether a particular phenotype of SMCs in the deep intima are primed to take up the retained lipoproteins concentrated in this region, or are converted to foam cells purely on the basis of being surrounded by these lipoproteins, requires further investigation. Rare macrophages may also migrate from the superficial layer to the middle of the intima at this stage (Guyton and Klemp, 1994) and contribute to the early foam cell formation. Some investigators suggest transmigration of adventitial monocytes at least into the arterial media (Maiellaro and Taylor, 2007). However, these observations have been reported in animal models in the context of restenosis after coronary angioplasty whose intervention completely strips the intima and leads to ruptures in the internal elastic lamina (Shi et al., 1996). To our knowledge, the sole study reporting migration of adventitial cells towards the intima in a context of native atherosclerosis is that of Eriksson, who showed images of migrating cells on the adventitial/intimal border in advanced lesions of apolipoprotein E (apoE) knockout (KO) mice (Eriksson, 2011). In contrast, there is no report of accumulation of adventitial macrophages in the intima of early human lesions.

In pathological intimal thickening (PIT), lipid-laden and non-lipid-laden macrophages may infiltrate more abundantly down to the middle of the intima (Otsuka et al., 2015; Nakagawa and Nakashima, 2018). Interestingly, following the initial population of the human pre-atherosclerotic intima with SMCs early in life, the number of SMCs remains stable from DIT to PIT, and there is no clear evidence of cell death or cell proliferation during this progression (Nakagawa and Nakashima, 2018). This also suggests that the migration rate of SMCs from the media to the intima is very low or non-existent in early lesions including in PIT (Nakagawa and Nakashima, 2018).

Regarding the relative contribution of SMCs and macrophages to the foam cell population, early studies suggested, based on the microscopic appearance of lesions and staining for SMCs and macrophages, that SMCs contribute the majority of these cells in fatty streaks and PIT (Stary, 1985, Stary, 1987; Katsuda et al., 1992). Allahverdian et al. (2014) attempted to quantitate this contribution specifically using immunohistochemistry. They estimated that, conservatively, SMCs are the source of at least 50% of foam cells in intermediate to advanced lesions based on costaining of smooth muscle actin (SMA) and Oil Red O following fixation to identify intracellular lipid localization. The contribution of SMCs to the total human foam cell population is potentially much higher, given that many intimal SMCs express low levels of or no SMA. In general, staining for macrophages including macrophage foam cells indicates the majority of these cells reside primarily in the immediate subendothelial region throughout human atherosclerosis but at later stages can migrate somewhat deeper into the upper-mid intima region where they are in immediate proximity with additional SMCs in the intima. Accurate identification of macrophages requires use of markers that are not known to be expressed by SMCs, such as CD45 (Allahverdian et al., 2014; Wang et al., 2019; Robichaud et al., 2022). Recent studies indicate that markers previously thought to be macrophage-specific, such as CD68, are expressed by a high percentage of intimal SMCs in both human and mouse lesions (Allahverdian et al., 2014; Feil et al., 2014), and should no longer be used to identify macrophages specifically in lesions.

**FIGURE 1:**
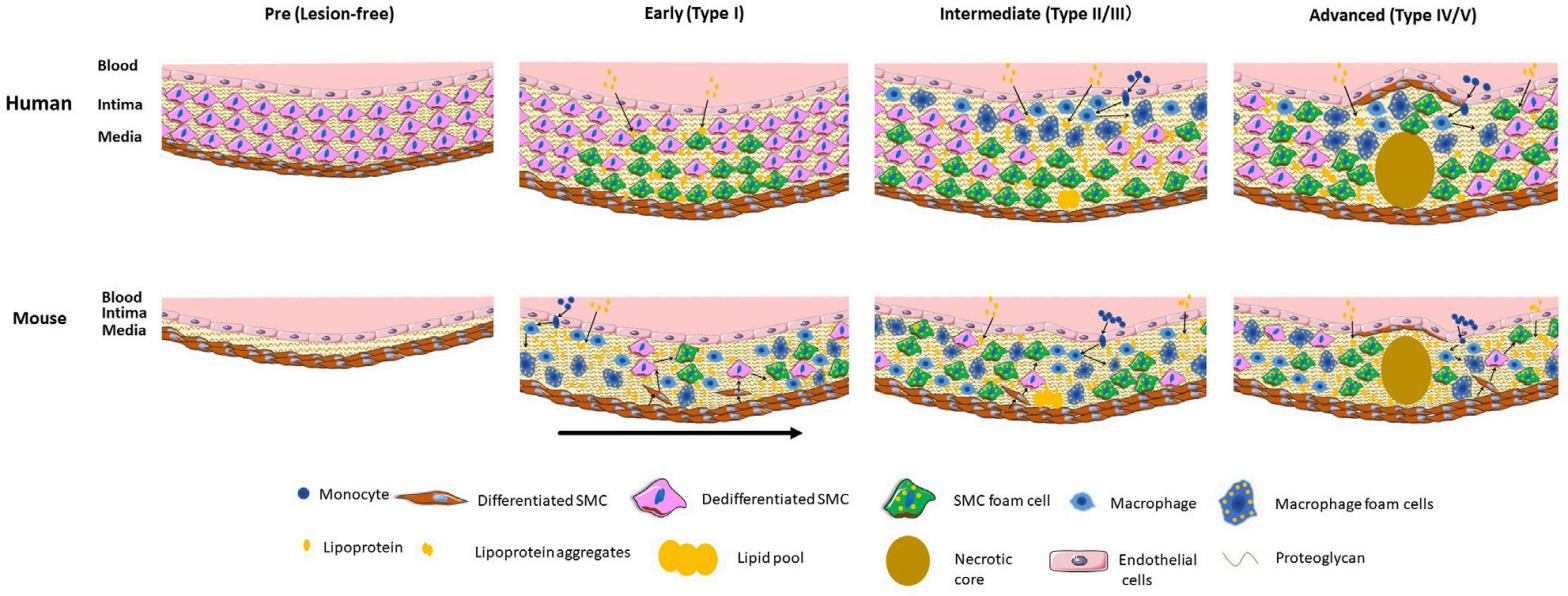
Stages and relative localization and contribution of SMCs and macrophage and their foam cells to human and mouse atherosclerosis. Human atherosclerosis is preceded by the formation of a thick intimal layer composed of SMCs and their secreted proteoglycans, known as diffuse intimal thickening. Atherogenic lipoproteins diffuse into the intima over decades and are deposited primarily in the deep intima, resulting in initial SMC foam cell formation. In intermediate lesions there is expansion of the SMC foam cell pool, and infiltration of circulating monocytes that differentiate to macrophages and take up lipids to become foam cells. In the advanced lesion a large necrotic core forms along with a fibrous cap of SMCs. Direct SMC-macrophage interactions occur primarily in the upper intima where most macrophages are located. Mouse atherosclerosis develops on a background of a healthy artery lacking diffuse intimal thickening. Severe dyslipidemia in mouse models induces endothelial inflammation and influx of lipids and monocytes that differentiate to macrophages and take up lipids to become foam cells. Cytokine-induced SMC migration occurs followed by proliferation and dedifferentiation of SMCs and uptake of lipids to become foam cells. In later early and more advanced mouse lesions SMCs make up the majority of foam cells. Direct as well as indirect macrophage-SMC interactions occur throughout the full thickness of the mouse lesion. The early mouse lesion shows different stages of development with the arrow indicating progression. In both humans and mice macrophage foam cells store cholesteryl ester droplets in the cytoplasm whereas in SMC foam cells the cholesteryl ester droplets appear mainly in lysosomes. Part of the figure was generated using Servier Medical Art, licensed under a Creative Commons Attribution 3.0 unported license.

The stages of development and relative contribution of SMCs and macrophages to the foam cell population in humans are indicated in the Figure 1.

## The Early and Direct Interaction Between SMCs and Macrophages in Mouse Lesions

### The Predominant Role of Macrophages in Early Atherosclerosis in Mice

The genetic manipulation of mice to generate atherosclerosis requires severe dyslipidemia, as achieved in models including apoE- and LDLR-KO mice on a C57BL/6 background who are also fed a high fat and cholesterol diet. The severe circulating dyslipidemia thus created itself represents a highly inflammatory environment for the vascular endothelium, in which immune cells such as monocytes/macrophages are rapidly recruited and have the predominant role in initiating atherogenesis (Swirski et al., 2006, Swirski et al., 2007). The absolute requirement for monocytes/macrophages to stimulate atherosclerosis in mice has been demonstrated by numerous investigators. Smith and co-workers generated apoE KO mice with homozygous or heterozygous mutation in the gene encoding macrophage colony-stimulating factor (MCSF) and fed them an ad libitum chow diet for 16 weeks. Double mutant (MCSF and apoE KO) mice showed an allele-dependent 85% decrease in atherosclerotic lesion areas compared to apoE KO mice expressing MCSF normally, despite a 3-fold increase in total cholesterol level (Smith et al., 1995). Similar findings were obtained on LDLR KO mice homozygous for MCSF mutation and fed an atherogenic diet, with complete absence of atherosclerotic lesions in the aortic arch after 16 weeks (Rajavashisth et al., 1998). Further strong evidence for the role of the immune system in atherogenesis in apoE KO mice comes from an elegant study from the group of Mallat. They combined inhibition of chemokine receptor type 2 (CCR2), C-X3-C motif chemokine receptor 1 (CX3CR1) and C-C motif chemokine receptor 5 (CCR5), three receptors involved in monocytosis of three specific subsets of monocytes, and compared the number of circulating monocytes and the size of lesions with WT mice. They reported a markedly reduced concentration of circulating monocytes accompanied with an almost complete abolition (>90% reduction) of atherosclerosis, despite elevated total cholesterol levels compared to WT mice (Combadière et al., 2008). These studies strongly attest to the predominant and excessive role of immune cells in particular of the myeloid lineage to induce atherosclerosis in mice, and contrast strongly with human atherosclerosis that is mainly induced by the initial SMC-driven retention of lipids in the intima.

### Early Macrophage Infiltration and Progressive Medial SMC Influx in Mouse Intima

As indicated above, SMCs, and DIT are not present in the intima of mouse aorta or other arteries, with SMCs only appearing following induction of atherogenesis. Although SMCs are strongly implicated in lipoprotein retention *via* proteoglycan secretion in humans, their absence in mouse intima is not accompanied by the absence of extracellular matrix proteins nor lipid retention. Indeed, in 3 week-old-only apoE KO mice fed a chow diet, Tamminen et al. (1999) reported the presence of individual lipid particles in a reticular network of branching filaments, thought to be proteoglycans, and containing numerous collagen fibrils. Subsequently, the first monocytes were observed in close association with endothelial cells in 5-week-old mice. At 9 weeks of age, the intima contained larger aggregated particles as well as more abundant subendothelial monocytes. This suggests that monocyte recruitment occurs secondarily to lipid retention but prior to SMC migration from the media. In a similar study, sporadic foam cells were observed in the aortic sinus of 11 week-old apoE KO mice fed a chow diet, and multilayered foam cell deposits as well as SMCs were observed in mice of 5–7 months of age (Reddick et al., 1994). Some resident and quiescent macrophages are also present in the lesion-free mouse lesions. However, their limited proliferative capacity and modest contribution in foam cells formation suggest a minor role in atherogenesis (Paulson et al., 2010; Williams et al., 2020; Hernandez et al., 2022). Nakashima also nicely described the development of atherosclerosis in mice with apoE deficiency and fed a Western diet (Nakashima et al., 1994). In their study, monocytes adherent to the endothelial surface and sporadic foam cells in the subendothelial space were observed in 6-week-old mice fed a Western Diet for 1 week. Visible fatty streaks lesions characterized by yellowish-white nodules appeared after 3 and 5 weeks of Western and chow diet, respectively. In mice fed a Western diet for 5–10 weeks, spindle-shaped cells thought to be SMCs were intertwined with foam cells or tended to form a cap around the lesion. By 10 weeks of Western diet (15 weeks of age), some lesions contained fibrous connective tissue with both necrotic cores and foam cells covered by a fibrous cap composed of SMCs.

Our lab has characterized foam cells in the aortic arch of non-lineage tracing and SMC-lineage tracing apoE KO mice using leukocyte-specific markers, neutral lipid staining and flow cytometry. In mice fed a Western diet for just 6 weeks, 70% of total foam cells were SMC- derived (Wang et al., 2019). This was very striking given the absence of SMCs in mouse intima at the onset of atherogenesis. This rapid progression is thought to be due to induction of SMC migration from the mouse arterial medial layer by cytokines secreted by inflamed endothelial cells and macrophages, followed by rapid proliferation and uptake of lipids by SMCs within the intima (Wang et al., 2019). Such a large contribution of SMCs to the foam cell content in mice had not previously been recognized, due to the loss of SMC markers by dedifferentiated SMCs, apparently more so in mice than in humans, and expression of macrophage markers by those same intimal SMCs (Feil et al., 2014; Shankman et al., 2015). Interestingly, in mice fed a lower fat chow diet, SMC-derived foam cells contributed 37% and 75% of the total foam cells at ages 27 and 57 weeks respectively, which emphasizes the gradually increasing role of SMCs in foam cell formation in mice (Wang et al., 2019). This is thought to be due to defects in cholesterol efflux from SMC compared to macrophage foam cells, as discussed below. A similar finding of the majority of foam cells being derived from SMCs was recently reported by the Ouimet group in mice injected with an adeno-associated viral (AAV) vector encoding gain-of-function PCSK9 (proprotein convertase subtilisin/kexin type 9) and fed a Western diet to induce hypercholesterolemia. The percentage of non-leukocyte foam cells increased from 60% to 76% between 6 and 25 weeks of Western diet, respectively (Robichaud et al., 2022). In the Wang et al. (2020) study, at 12 weeks of Western diet, both macrophages and SMCs appeared intermingled throughout the whole thickened intima. These studies clearly indicate that macrophage infiltration is followed rapidly by the influx of SMCs from the surrounding media in mice. As macrophages are present in the entire intima following lipid infiltration, migrating SMCs from the media are in direct contact with macrophages as soon as they arrive in the intima. As such, there are marked differences in the localization of SMCs and macrophages in human when compared to mouse lesions, with human lesions maintaining relatively sequestered locations of these two cell types until more advanced lesion stages, whereas in mice macrophages and SMCs are intermingled from very early stages after SMCs soon begin to migrate into the intima.

The stages of development and the relative contribution of macrophages and SMCs to the foam cell population in mice are indicated in the Figure 1.

### Smooth Muscle Cell—Macrophage Interactions Leading to Foam Cell Formation in Atherosclerosis

While arterial SMCs in humans and mice can express macrophage markers as they dedifferentiate from contractile to synthetic, intimal-type SMCs (Rong et al., 2003; Allahverdian et al., 2014; Feil et al., 2014; Shankman et al., 2015), they never achieve full macrophage function, exhibiting at most 25% of the ability of macrophages to perform phagocytosis and efferocytosis when dedifferentiated (Vengrenyuk et al., 2015). SMCs do, however, like macrophages, express a host of scavenger receptors that can, upon exposure over years to retained lipoproteins in the human intima, or even weeks in the mouse intima, take up these lipoproteins to become foam cells. In the following section, we outline the key mechanisms known to allow macrophages and SMCs to become foam cells, and interactions between these cells that promote foam cell formation by the reciprocal cell type. A Summary of the mediators of lipoprotein uptake promoting foam cell formation by macrophages and SMCs is presented in Table 1.

**TABLE 1:** Summary of major lipoprotein uptake mediators driving foam cell formation in macrophages and SMCs.

Cell type	Mediators	Lipoprotein ligands	SR activators
Macrophages	SRA	AcLDL and oxLDL (Kunjathoor et al., 2002b)	MAPK signaling, hyperglycemia (Ben et al., 2009; Zhu et al., 2011)
	CD36	OxLDL (Boullier et al., 2000; Podrez et al., 2002; Nicholson, (2004)	PPARγ, IL-4, Hyperglycemia (Tontonoz et al., 1998; Feng et al., 2000; Nicholson, 2004)
	LOX1	OxLDL (Pirillo et al., 2013)	OxLDL, inflammatory cytokines (Kattoor et al., 2019)
	LRP1	AgLDL (Llorente-Cortés et al., 2007)	SREBP inhibition
	Macropinocytosis/exophagy	Native or modified LDL (Jones et al., 2000; Kruth et al., 2005)	TLR4 (Kruth et al., 2005; Kruth, 2013)
	Proteoglycan	LDL (Ng et al., 2021)	Inflammatory stimuli or hypoxia stress (Uhlin-Hansen et al., 1993; Asplund et al., 2011)
SMCs	SRA	AcLDL and oxLDL (Mietus-Snyder et al., 2000)	OxLDL, growth factors from macrophages and platelets, IFNγ, MAPK signaling (Gong and Pitas, 1995; Li et al., 1995)
	CD36	OxLDL (Zingg et al., 2002)	PPARγ, VEGF, MAPK signaling, hyperglycemia, oxLDL (Matsumoto et al., 2000; Zingg et al., 2000; Xue et al., 2010; Schlich et al., 2015)
	LOX1	OxLDL (Aoyama et al., 2000)	OxLDL, inflammatory cytokines (Aoyama et al., 2000; Hofnagel et al., 2004)
	LRP1	AgLDL (Llorente-Cortés et al., 2000; Llorente-Cortés et al., 2002; Llorente-Cortés et al., 2006)	P2Y2 receptor (Dissmore et al., 2016)
	Proteoglycan	LDL (Nakashima et al., 2007; Neufeld et al., 2014)	oxLDL, TGFβ, PDGF, SMC dedifferentiation (Camejo et al., 1993; Chang et al., 2000; Doran et al., 2008; Bennett et al., 2016)

## Mediators and Regulation of Macrophage Foam Cell Formation

While circulating levels of LDL-C are tightly associated with atherogenesis and ischemic vascular disease (Yusuf et al., 2004), native LDL is a poor inducer of SMC and macrophage foam cell formation because its main receptor, the LDLR, is downregulated in response to cholesterol loading of cells (Goldstein and Brown, 1977; Goldstein et al., 1977; Brown and Goldstein, 1983). Excess cholesterol derived from LDL inhibits the proteolytic cleavage of sterol regulatory element-binding protein (SREBP) in the endoplasmic reticulum and therefore inhibits migration of the SREBP fragment to the nucleus, which reduces the transcription of the *LDLR* gene (Brown and Goldstein, 1999; Goldstein and Brown, 2009). The key ligands thought to be responsible for foam cell formation *in vivo* are aggregated and oxidized forms of LDL and other apoB-containing lipoproteins (Steinbrecher and Lougheed, 1992). Previous research has indicated the predominant receptors that drive unregulated uptake of these modified apoB-containing lipoproteins are scavenger receptors (SRs) (Brown et al., 1979). SRs are thought to be beneficial in their ability to clear modified lipoprotein accumulation in the artery wall, but the unregulated uptake of these lipoproteins by SRs leads to overaccumulation of lipoprotein-derived cholesteryl esters, the biochemical hallmark of foam cells. In macrophages, this results in cholesteryl ester droplet accumulation in the cytoplasm with the droplets imparting a “foamy” appearance; excess macrophage foam cells promote inflammation and instability of the plaque. As noted below, SMC foam cells appear to store their excess cholesteryl esters in lysosomes rather than in the cytoplasm, due to relative deficiency of lysosomal acid lipase (LAL) (Dubland et al., 2021) (Figure 1).

### Scavenger Receptor-Dependent Macrophage Foam Cell Development

There are 12 classes of scavenger receptors identified on membrane surfaces, which are class A-L grouped based on their structural characteristics (PrabhuDas et al., 2017). Among the 12 classes, the class A scavenger receptors A I/II and the class B receptor CD36 are the most studied and believed to be the major receptors taking up modified lipoproteins (Kunjathoor et al., 2002b). Scavenger receptors A I/II (SRA) share a homotrimeric structure composed of a collagen-like domain and a c-type lectin domain. Despite having some structural differences, the two receptors’ functions and regulations are similar (Moore and Freeman, 2006). It was reported that in mouse macrophages SRA was responsible for 70% of uptake of the acetylated LDL (acLDL) and 40% of the uptake of oxidized LDL (oxLDL) based on intracellular LDL degradation (Kunjathoor et al., 2002b). A significant reduction (60%–80%) in atherosclerotic lesion area was observed in mouse models with SRA deficiency, confirming this *in vitro* observation (Babaev et al., 2000; Nicholson, 2004). The atherogenic role of SRA was enhanced in hyperglycemic conditions, which upregulates SRA expression (Fukuhara-Takaki et al., 2005). Using mouse RAW264.7 macrophages, it was shown that SRA-mediated endocytosis activated extracellular signal-regulated kinase (ERK) signaling and c-Jun N-terminal kinase (JNK) signaling (Zhu et al., 2011), which are part of the mitogen-activated protein kinase (MAPK) pathway (Ben et al., 2009; Zhu et al., 2011). At the same time, activation of the JNK and ERK signaling pathways cooperatively enhanced SRA transcription factors binding to the promoter, driving a positive feedback loop to enhance SRA receptor gene expression on the cell surface (Moulton et al., 1994). Further studies confirmed that inhibition of protein kinase C (PKC), a kinase in the ERK signaling pathway, and c-Jun, the final transcription factor of JNK signaling, downregulated SRA expression and reduced lipoprotein uptake (Akeson et al., 1991; Moulton et al., 1992).

CD36 belongs to the class B scavenger receptor family, which traverse the plasma membrane twice to form a loop with a heavily glycosylated extracellular portion and an intracellular tail to transmit the signal (Moore and Freeman, 2006). Similar to SRA, CD36 also promotes modified lipoprotein uptake and shows a high affinity to oxidized lipoproteins *in vitro* and in mice (Boullier et al., 2000; Podrez et al., 2002; Nicholson, 2004). CD36 regulation is mediated by peroxisome proliferator-activated receptor (PPARγ), which is upregulated in response to exposure to oxLDL (Tontonoz et al., 1998; Feng et al., 2000; Nicholson, 2004). Similar to SRA, hyperglycemia increases CD36 mRNA abundance and translational efficiency, leading to more scavenger receptors expressed on the membrane (Griffin et al., 2001). Though CD36 and SRA receptors play significant roles in taking up modified lipoproteins, previous research showed macrophages can still become foam cells without CD36 and SRA, indicating there are numerous mediators of foam cell development (Sakr et al., 2001; Kunjathoor et al., 2002b; Manning-Tobin et al., 2009). For example, it was observed that lectin-like oxidized low-density lipoprotein receptor-1 (LOX1) contributed to oxLDL uptake in macrophages, which was upregulated with increased oxidative stress or in the presence of proinflammatory cytokines (Pirillo et al., 2013; Kattoor et al., 2019). Another example is LDLR-related protein-1 (LRP1), knockdown of which reduced cholesteryl ester accumulation from aggregated LDL (agLDL) in macrophages by 95%. Interestingly, the receptor was subjected to negative regulation by sterol regulatory element-binding protein 1 (SREBP1) (Llorente- Cortés et al., 2007). Thus, in hypercholesterolemic conditions, the expression of LRP1 likely remains high as SREBP1 is not delivered to the nucleus, confirming the observation that the receptor can play a significant role in agLDL uptake and foam cell formation. Scavenger receptor B1 (SRB1) usually functions as a SR driving cholesterol efflux (Moore and Freeman, 2006; Hu et al., 2013), but research by Marsche et al. showed that SRBI also mediates the uptake of hypochlorite modified-LDL and HDL, which promoted lipid accumulation while reducing cholesterol efflux, leading to foam cell development (Marsche et al., 2002; Marsche et al., 2003). Other receptors that can take up modified lipoproteins include scavenger receptor expressed by endothelial cells-I (SREC-I) (Shimaoka et al., 2000), C-X-C motif chemokine ligand 16 (CXCL16) (Barlic et al., 2006; Sheikine and Sirsjö, 2008), CD68 (Ramprasad et al., 1996), and CD146 (Luo et al., 2017), which are less well characterized compared to the ones described (Moore and Freeman, 2006; Kzhyshkowska et al., 2012; Checkouri et al., 2021).

### Scavenger Receptor-Independent Macrophage Foam Cell Formation

Further research has described mechanisms of macrophage foam cell development independent of lipoprotein receptors. Human-monocyte derived macrophages can take up LDL *via* fluid-phase pinocytosis, which is a receptor-independent uptake mechanism whereby LDL is taken up *via* large vacuoles through micropinocytosis, or *via* small vesicles through micropinocytosis. Though both pathways are equally important in LDL uptake, we have very limited knowledge of the role of micropinocytosis in cholesterol uptake and therefore will focus on macropinocytosis (Kruth, 2013). Macropinocytosis was shown to drive foam cell formation with native LDL or modified LDL (Jones et al., 2000; Kruth et al., 2002, Kruth et al., 2005). It was observed that human monocyte-derived macrophages take up lipoproteins along with fluid in a sealed macropinosome (Kruth et al., 2002, Kruth et al., 2005; Barthwal et al., 2013; Doodnauth et al., 2019). This uptake increased with increased extracellular lipoprotein concentrations and was subjected to regulation by actin, microtubules, vacuolar-type H + ATPase (V-ATPase), phosphoinositide 3- kinase (PI3K), and spleen tyrosine kinase (SYR) in a toll-like receptor 4 (TLR4) dependent manner (Kruth et al., 2005; Kruth, 2013). Interestingly, a similar mechanism was observed to upregulate exophagy, or extracellular degradation of large elements such as agLDL. Using the mouse macrophages*,* it was observed that agLDL triggered activation of PI3K and SYR-dependent TLR4 signaling in the macrophage. TLR4 signaling triggered actin polymerization and lysosomal exocytosis to form an acidic lysosomal synapse in the extracellular environment, where lysosomal acid lipase (LAL) degraded the aggregate extracellularly into free cholesterol (Haka et al., 2009; Singh et al., 2016, Singh et al., 2020). Free cholesterol can then be taken up by macrophages to drive foam cell formation. More research is necessary to identify if macropinocytosis is linked to exophagy or if these are two independent pathways subjected to similar regulation.

Proteoglycans are complex macromolecules consisting of the core protein to which linear negatively charged glycosaminoglycan (GAG) molecules are covalently linked (Wight, 2018; Allahverdian et al., 2021). It was observed that the proteoglycan content increases dramatically during early atherosclerosis, which was predominantly composed of versican, biglycan, and decorin in humans (Nakashima et al., 2007, Nakashima et al., 2008; Wight, 2018) and biglycan and perlecan in mice (Kunjathoor et al., 2002a). As noted above, proteoglycans retain apoB- carrying lipoproteins in the subendothelial space through ionic interactions, specifically through the positively charged arginine and lysine residues on apoB and negatively charged sulfates on proteoglycans (Borén et al., 2000; Nakashima et al., 2007; Wight, 2018; Allahverdian et al., 2021). Within the intimal space, retained lipoproteins can undergo enzymatic or chemical modifications, which increases the atherogenicity and subendothelial retention of the lipoproteins (Aviram, 1993; Tabas, 1999). Simultaneously, the presence of proteoglycans can cause irreversible structural disruption of lipoproteins (Camejo et al., 1998). *In vitro* structural analysis and oxidation experiments showed that lipoproteins exposed to chondroitin sulfate had increased exposure of arginine and lysine residues and had greater chance to undergo oxidation, thereby increasing the binding affinity to scavenger receptors on SMCs and macrophages (Hurt-Camejo et al., 1992; Vijayagopal et al., 1993; Ismail et al., 1994; Krettek et al., 1997).

Though SMCs are the major source of proteoglycan secretion in the intima, macrophages can also secrete proteoglycans that contribute to lipoprotein retention (Kolset and Gallagher, 1990; Wight, 2018; Ng et al., 2021). For example, macrophages synthesize chondroitin sulfate proteoglycans, which connect atherogenic LDL and SRA in proximity, promoting more lipoprotein uptake and foam cell development (Christner, 1988; Santiago-García et al., 2003). Analysis of macrophage conditioned medium showed that macrophage colony-stimulating factor (MCSF) could complex with proteoglycans when secreted by macrophages, which promoted monocyte differentiation and lipoprotein retention at the same time, contributing to foam cell development (Chang et al., 1998)*.* Recently, Ng and colleagues also identified that perlecan was the major proteoglycan secreted by human macrophages involved in LDL retention, though perlecan was also suggested to be low in human lesions (Tran et al., 2007; Ng et al., 2021). It was observed that proteoglycan synthesis by macrophages was upregulated when macrophages received inflammatory stimuli or were under hypoxia stress. (Uhlin-Hansen et al., 1993; Asplund et al., 2011).

### Influence of Macrophage Polarization on Foam Cell Development

Macrophage phenotype is highly plastic, including levels of SR expression (Yang et al., 2020). For example, interferon-gamma (IFNγ) is a potent cytokine inducing a proinflammatory response in human monocyte-derived macrophages. Proinflammatory macrophages (M1) were shown to have lower CD36 and SRA expression and therefore had reduced uptake of modified lipoproteins (Geng and Hansson, 1992; Zingg et al., 2000; Chu et al., 2013; Murray et al., 2014). In contrast, alternatively activated macrophages (M2), which were polarized by interleukin-4 (IL-4) *in vitro* (Murray et al., 2014), had greater CD36 expression and uptake of modified lipoproteins (Yesner et al., 1996; Chu et al., 2013; Murray et al., 2014). Alternatively activated macrophages also had higher macropinocytosis activity *in vitro*, promoting lipoprotein phagocytosis within the cells (Redka et al., 2018). Different macrophage phenotypes may also secrete different proteoglycans, modulating extracellular lipoprotein retention (Ng et al., 2021). It should be noted that the polarization impact described here focuses on human monocyte-derived macrophages, and the effect may vary depending on the macrophage origin and type (Geng and Hansson, 1992; Fitzgerald et al., 2000).

## Mediators and Regulation of SMC Foam Cell Formation

In recent years, since the introduction of mouse models of atherosclerosis, foam cells have been thought to mostly be derived from macrophages, and to have major importance especially in areas where plaques are prone to rupture, such as the fibrous cap or shoulder regions (Ross, 1995; Libby et al., 2011; Bennett et al., 2016; Chistiakov et al., 2017). While macrophage foam cells are undoubtedly important for plaque instability and rupture, recent studies now suggest a major role of SMCs as a source of foam cells in human and mouse atherosclerosis, and as likely predictors of the development and regression of atherosclerotic lesions. Immunostaining of early atherosclerotic lesions showed a large number of SMC foam cells in the deep intima in early atherosclerosis and suggested SMCs are an important source for foam cells (Katsuda et al., 1992; Nakashima et al., 2007). Recent studies using early and advanced coronary artery lesions from mice and humans have indeed confirmed that the majority of foam cells in both early and advanced lesions are SMC-derived (Allahverdian et al., 2012; Wang et al., 2019).

### Scavenger Receptor-Dependent SMC Foam Cell Development

Intima SMCs have higher scavenger receptor activity than medial SMCs, likely due to their innate phenotype and possibly due to exposure to diverse growth factors secreted by macrophages and platelets (Gong and Pitas, 1995). Growth factors can upregulate SRA synergistically up to 7-fold *in vitro* (Gong and Pitas, 1995; Li et al., 1995). Using an *in vitro* SMC model, studies from the Pitas group showed that the presence of oxLDL can activate tyrosine-protein kinase, triggering MAPK signaling cascade and upregulating the c-Jun transcription factor. C-Jun then increased SRA and cyclooxygenase-2 expressions, contributing to acLDL uptake and foam cell formation (Mietus-Snyder et al., 1997, Mietus-Snyder et al., 2000). However, the importance of SRA in SMC foam cells is questionable, as SRA was reported to be dispensable in driving SMC foam cell development (Luechtenborg et al., 2008) and was primarily associated with macrophages in atherosclerotic lesions (Pryma et al., 2019). Interestingly, the regulation of SRA in SMCs appears to be opposite compared to in macrophages. For example, though IFNγ is a negative regulator for macrophage SRA, it can increase SRA mRNA expression and its acLDL degradation activity in cultured SMCs (Li et al., 1995).

CD36 is another scavenger receptor expressed in human SMCs, reported to be responsible for 80% of oxLDL uptake and the uptake of free fatty acids (Zingg et al., 2002; Ma et al., 2011). CD36 is primarily upregulated by its ligand oxLDL, which activates PPARγ to upregulate CD36 transcription (Matsumoto et al., 2000; Zingg et al., 2000). Tyrosine kinase inhibition downregulates CD36 *in vitro* and reduces oxLDL uptake in SMCs (Lin et al., 2015), suggesting that the MAPK signaling pathway is also involved in CD36 positive regulation. Endothelial-1, which is an endogenous factor stimulating SMC proliferation, was shown to reduce CD36 expression in a tyrosine kinase-dependent manner (Kwok et al., 2007). In contrast, the growth factor VEGF that promotes SMC proliferation can increase CD36 expression, suggesting different growth factors might regulate CD36 differently (Schlich et al., 2015). Interestingly, it was observed that CD36 expression on SMCs is upregulated in diabetic patients, triggered by hyperglycemia condition and oxLDL exposure, leading to increased cholesterol influx and SMC foam cell development (Xue et al., 2010; Navas-Madroñal et al., 2020).

Besides the classical SRs, SMCs also express LOX1 and LRP1 when exposed to modified lipoproteins (Pryma et al., 2019; Checkouri et al., 2021). Llorente-Cortés and colleagues showed that LRP1 was responsible for 80% uptake of the agLDL and 65% of versican-modified LDL *in vitro* (Llorente-Cortés et al., 2000; Llorente-Cortés et al., 2002; Llorente-Cortés et al., 2006). Additional research suggested that LRP1 was subjected to P2Y2 receptor regulation in SMCs, a receptor triggering rearrangement of the actin cytoskeleton and cell motility (Dissmore et al., 2016). Similar to macrophages, LOX1 is also present in SMCs and is upregulated in the presence of oxLDL (Aoyama et al., 2000) and inflammatory cytokines (Hofnagel et al., 2004). C-X-C-motif chemokine ligand 16 (CXCL16) (Wågsäter et al., 2004) and receptor for the advanced glycation end products (RAGE) (Bao et al., 2020) were identified to act as scavenger receptors to promote SMC foam cell formation, but further investigation is required to determine the precise role and regulation of the receptors in atherosclerosis. Intriguingly, it was shown *in vitro* that SMCs can ingest lipoproteins modified by trypsin and cholesteryl ester hydrolase *via* calcium-dependent macropinocytosis, but the detailed mechanism and the clinical relevance of macropinocytosis remains to be explored (Chellan et al., 2016).

### Scavenger Receptor-Independent SMC Foam Cell Development

As the predominant cell source of proteoglycans in the vascular wall (Wight, 1985; Nigro et al., 2005), SMCs are responsible for the increase in proteoglycan content in early atherosclerosis (Wight, 2018). Among the proteoglycans secreted, biglycan and decorin were shown to have the highest binding affinity towards LDL and were enriched in atherosclerotic lesions in the deep intima, suggesting both proteoglycans were major players in LDL retention in this location (Riessen et al., 1994; O’Brien et al., 1998; Nakashima et al., 2007; Neufeld et al., 2014). Perlecan was shown to be important in mice in promoting lipoprotein retention and increasing vascular permeability but is reduced in human atherosclerotic lesions, likely due to species difference (Tran-Lundmark et al., 2008; Allahverdian et al., 2021). The synthesis of proteoglycans is also governed by lipoproteins that SMCs are exposed to. Using monkey SMCs as an *in vitro* model, oxLDL was shown to induce elongation of glycosaminoglycan chains of biglycan, decorin, and versican, which was not observed with native LDL and could induce 30%–50% more total proteoglycan synthesis compared to native LDL (Camejo et al., 1993; Chang et al., 2000; Doran et al., 2008). In addition, the presence of growth factors that promote SMC proliferation, such as PDGF and TGFβ, promoted the synthesis of versican and biglycan in SMCs in an NF-kb—dependent manner (Schönherr et al., 1993; Chang et al., 2000; Basatemur et al., 2019), contributing to the lipoprotein retention.

Intima SMCs may transit into a synthetic state with a gain of macrophage markers such as CD68 in culture and in atherosclerotic lesions (Bennett et al., 2016; Basatemur et al., 2019). Synthetic SMCs have increased proteoglycan synthesis (Bennett et al., 2016) and phagocytosis activity (Sandison et al., 2016), contributing to excess cholesterol retention and uptake. Interestingly, it was observed that cholesterol uptake (Rong et al., 2003) and proteoglycans in the subendothelial space (Roy et al., 2002; Allahverdian et al., 2021) could be positive feedback regulators that contributed to SMC dedifferentiation *in vitro*, initiating a vicious cycle in driving more lipid uptake in SMCs.

## SMC-Macrophage Interactions in Foam Cell Development

Immunohistochemistry studies on human and mouse atherosclerotic lesions have indicated macrophages and SMCs are in proximity in early and intermediate stages of atherosclerosis, depending on the species, suggesting that interactions between macrophages and SMCs are important in atherogenesis (Nakashima et al., 2007; Wang et al., 2019). Numerous *in vitro* studies have found that interactions between macrophages and SMCs can promote SMC proliferation (Zhang et al., 1993; Chen et al., 2014), proteoglycan/matrix metalloproteinase synthesis (Edwards et al., 1990; Zhu et al., 2000), migration (Niu et al., 2016), and dedifferentiation into the synthetic state (Beck-Joseph and Lehoux, 2021). In reverse, SMCs can also contribute to monocyte adhesion, recruitment, and survival (Doran et al., 2008; Beck-Joseph and Lehoux, 2021). SMC-macrophage interactions can also heighten inflammation in both cell types and can transform SMCs into a macrophage-like phenotype (Butoi et al., 2014). For this review, we will specifically focus on the interactions between SMCs and macrophages leading to foam cell development in atherosclerosis. A summary of the potential SMC-macrophage interactions in foam cell development is presented in Table 2.

**TABLE 2:** Summary of potential SMC-macrophages interactions in foam cell development.


Direct interaction	Macrophage effect on SMCs
	• Increase SMC phagocytic activity (Vijayagopal and Glancy, 1996)
	• Delivery of macrophage cholesterol into SMCs (Weinert et al., 2013; He et al., 2020) SMC effect on macrophages
	• Increase CD36 expression on monocytes
Indirect interaction	Macrophage effect on SMCs
	• Increase cholesterol uptake and degradation (Aviram, 1989; Nishide et al., 1992; Stein et al., 1994; Mietus-Snyder et al., 2000; Wang et al., 2019)
	• Transfer cholesterol directly to SMCs (Wolfbauer et al., 1986; He et al., 2018; Hu et al., 2019)
	• Increase LAL activity (Dubland et al., 2021)
	SMC effect on macrophages
	• Increase cholesterol degradation (Aviram, 1989)
	• Increase CD36 expression on monocytes (Cai et al., 2004)
Limitations	• Limited ability to mimic artery wall milieu (Zuniga et al., 2014; Beck-Joseph and Lehoux, 2021)
	• Inconsistent SMC and macrophage origins and phenotypes (Murray et al., 2014; Allahverdian et al., 2018)

### Cell-Cell Interactions Between SMCs and Macrophages

*In vitro* models showed that the direct contact between macrophages and SMCs could promote increased foam cell development in SMCs but the mechanism causing the increase in lipoprotein uptake was not clear.Vijayagopal and Glancy, (1996) reported that the presence of macrophages could increase SMC phagocytic activity and therefore lipoprotein uptake. However, Hu et al. and Weinert et al. (2013) suggested that macrophages can deliver cytosolic and lysosomal cholesterol into SMCs when in close proximity, possibly through cell-cell membrane interactions (He et al., 2020). Though we did not find an *in vitro* study assessing the role of direct cell contact in macrophage foam cell development, it was observed *in vitro* that direct or indirect coculture of human SMCs with monocytes could lead to an increase in CD36 scavenger receptor expression and oxLDL uptake by monocytes. It was shown that monocyte chemoattractant protein-1 (MCP-1) secreted by SMCs was responsible for contact-independent CD36 upregulation in monocytes, while vascular adhesion molecule-1 (VCAM-1) on SMCs was responsible for contact-dependent CD36 upregulation in monocytes (Cai et al., 2004).

### Indirect Interactions Between SMCs and Macrophages

Previous studies also assessed the indirect interactions between macrophages and SMCs in foam cell development. By measuring the LDL degradation rate, Aviram’s early study showed that conditioned media from macrophages doubled the LDL degradation rate within SMCs while SMC’s conditioned media enhanced macrophage LDL degradation by 15%, suggesting that the conditioned media of macrophages and SMCs both promote foam cell development in the other cell type (Aviram, 1989). Similarly, Stein et al. (1994) and Nishide et al. (1992) showed that conditioned media from macrophages triggered a ten-fold increase in cholesterol uptake by SMCs compared to control SMCs. Component analysis by Stein et al. (1994) identified that lipoprotein lipase, apoE, and proteoglycans likely played key roles in lipoprotein adhesion to the SMC surface and therefore in promoting lipoprotein uptake. Using a conditioned media approach, previous research observed that macrophages could release cholesterol-rich particles that can be taken up by SMCs through phagocytosis and promote SMC foam cell formation (Wolfbauer et al., 1986; He et al., 2018; Hu et al., 2019). Similarly, Mietus-Synder et al. (2000) showed that incubation of SMCs with THP-1 macrophage-conditioned media also showed a 25–30-fold increase in the uptake of Dil-labeled acLDL by SMCs, which was not observed if there was no serum or with lipid poor serum in the conditioned media. Using a collagen gel system model, a recent study done by Wang et al. (2019) observed that the presence of mouse macrophage led mouse SMCs to have a 5-fold increase in agLDL uptake without having direct cell-cell contact, confirming the early observations.

In addition, using the conditioned media approach, several articles showed that macrophages might promote cholesterol esterification within SMCs while promoting lipoprotein uptake. Stein et al. showed that after 24 h incubation with macrophage conditioned media, SMCs had a 3-4-fold increase in cholesteryl ester content compared to with SMCs alone, which could be enhanced further by another 3-fold if the conditioned media was from the macrophage foam cells (Stein et al., 1981, Stein et al., 1993, Stein et al., 1994). Recent research by Dubland et al.(2021) also showed that the conditioned media from macrophages could lead to a 2.8-fold increase in SMC LAL activity, indicating the ability of macrophages to influence LAL-dependent cholesterol metabolism in SMCs (discussed further below).

### *In vitro* Models for the Study of SMCs-Macrophages Interactions in Atherosclerosis

Currently, most *in vitro* models utilize direct cell contact models to study direct cell-cell interactions and conditioned media or transwell models to study secreted soluble mediator effects. While the models are relatively simple to set up, they have several limitations. Firstly, most *in vitro* models are 2-dimensional (2D) and therefore cannot capture fully the artery wall structure and dynamics observed *in vivo*, such as blood flow and extracellular matrix (Zuniga et al., 2014; Beck-Joseph and Lehoux, 2021). Previous research attempted to mimic the artery wall using a gel scaffold model, but the composition of the scaffold in healthy and diseased states remains to be determined to ensure the model’s physiological relevance (Sukhova et al., 1999; Wight, 2018). Recent engineered tissue designs include microfabricated vessels with biomaterials to mimic the functional blood vessel *in vivo*, but the model remains to be tested to assess its applicability in studying cell-cell interactions and foam cell formation (Moses et al., 2021). Secondly, as different studies utilized cells from different origins, species differences may contribute to the discrepancies observed among studies. The plasticities of both cell types can add a layer of complexity when comparing different co-culture studies (Murray et al., 2014; Allahverdian et al., 2018). Therefore, future studies should characterize the cell phenotypes before performing interaction studies to ensure the reproducibility of the results.

## Potential Role of Macrophage-SMC Interactions in Promoting Cholesterol Efflux From Foam Cells, and Potential Therapeutic Interventions

Cholesterol removal from foam cells is dependent on external factors such as reducing the further influx of atherogenic lipoproteins into the artery wall through the use of LDL-lowering medications, and promoting intracellular cholesterol efflux pathways. The latter include secretion of cellular cholesterol to form HDL particles by upregulation of the cholesterol exporter protein ATP-binding cassette transporter A1 (ABCA1), achieved through the action of intracellular oxysterols binding to liver X receptor (LXR) nuclear receptors on the promoter region of the *ABCA1* gene in the nucleus (Oram and Heinecke, 2005). Additional cholesterol efflux pathways include ABCG1, also upregulated by intracellular oxysterols, SR-BI, and passive diffusion of cholesterol from the plasma membrane to preformed HDL particles in the extracellular space (Phillips, 2014). We previously determined that ABCA1 expression is low in arterial SMCs when compared to macrophages in both humans and mice (Allahverdian et al., 2014; Wang et al., 2019); in human SMCs we have also determined their expression of sterol-27-hydroxylase, responsible for production of 27-hydroxycholesterol, the key oxysterol required for upregulation of ABCA1 in the artery wall via LXRα (Björkhem et al., 1994), as well as LXRα itself, are also low when compared to macrophages (Dubland et al., 2021). As such, arterial SMCs have a number of potential reasons for impaired upregulation of ABCA1.

Perhaps most remarkably, arterial SMCs of both humans and mice exhibit inherently low expression of *Lipa*, responsible for LAL expression, when compared to macrophages in both species (Dubland et al., 2021). This results in sequestration of lipoprotein-derived cholesteryl esters within the lysosomes of SMCs, rather than in the cytoplasm as in macrophage foam cells (Figure 1). Addition of conditioned medium from macrophages, which contains secreted LAL, or exogenous LAL, is, however, able to correct cholesterol efflux in SMC foam cells, despite lack of a further increase in ABCA1 expression in these cells (Dubland et al., 2021). As such, *in vivo*, it is possible that macrophage-secreted LAL may be taken up and be able to promote cholesterol efflux from nearby SMC foam cells, depending on the proximity of the surrounding macrophages and the ability of secreted LAL to diffuse within the plaque interstitial fluid.

Other potential therapeutics aimed specifically at influencing SMC-macrophage interactions could play an important role in reducing atherosclerosis or inducing its regression. An example is compound NBI-74330, an antagonist for C-X-C motif chemokine receptor 3 (CXCR3) on the surface of macrophages, which blocks SMC-secreted ligand C-X-C motif chemokine ligand 10 (CXCL10) from activating CXCR3 to drive monocyte retention and inflammation (van Wanrooij et al., 2008; van den Borne et al., 2014). *In vivo,* NBI-74330 treatment led to a significant reduction in mouse lesion area (van Wanrooij et al., 2008). An additional target influencing both SMC and macrophage foam cell formation is the lipoprotein-proteoglycan interaction responsible for arterial lipoprotein retention. Antibody chp3R99 has been developed to recognize sulfated glycosaminoglycans, blockage of which inhibits lipoprotein binding and reduces atherosclerotic lesion formation in the rabbit (Soto et al., 2012). Similarly, removing chondroitin sulfate from versican was also shown to reduce lipid retention and monocyte recruitment in a rabbit model (Merrilees et al., 2011). Further investigations are required to determine whether targeting interactions between SMCs and macrophages, as well as recruitment of each cell type to and retention of lipoproteins in the intima, will come to fruition as novel treatments to prevent residual risk from atherosclerosis not addressed by currently available medications.

## Conclusion

In both humans and mice, the relative contribution of SMCs and macrophages, the timing of their appearance, and their relative proximity to each other and deposited lipids in the artery wall are all key determinants of the initiation and progression of atherosclerosis. A clearer understanding of these steps and the interactions between SMCs and macrophages that influence foam cell development and regression are likely to provide fertile ground in the years ahead for development of novel treatments that will reduce ischemic vascular disease beyond what is possible with currently available treatments.
